# “Massa de Malagueta”: tradition with a twist

**DOI:** 10.1080/07853890.2021.1896073

**Published:** 2021-09-28

**Authors:** Lia Correia, Inês Mendes, Sofia Ortiz, Renata Ramalho, Paula Pereira

**Affiliations:** a Instituto Universitário Egas Moniz (IUEM), Egas Moniz Cooperativa de Ensino Superior, Caparica, Portugal; b Centro de Investigação Interdisciplinar Egas Moniz (CiiEM), Egas Moniz Cooperativa de Ensino Superior, Caparica, Portugal; c Grupo de Estudos em Nutrição Aplicada (GENA), Instituto Universitário Egas Moniz (IUEM), Egas Moniz Cooperativa de Ensino Superior, Caparica, Portugal

## Abstract

**Introduction:**

OMS estimates that, in 2010, approximately 600 million people had hypertension (HT) globally. The prevalence of HT in Portugal is between 30 ando 45% and in the Azores is 33.6%. The high salt chilli sauce “Massa de Malagueta (MM)”, widely used in this archipelago, may contribute to this prevalence since excess dietary sodium predisposes to high blood pressure.

**Objectives:**

Our aims were to reformulate the recipe of MM into a healthier version (lower salt, better ingredients), to determine its organoleptic acceptability, and total antioxidant capacity (TAC) in comparison to the traditional recipe.

**Methods:**

Traditional version was designed sample A. The healthier version (sample B) was prepared by combining pepper, shredded tomato, dried tomato, garlic, Indian saffron, iodised salt (2.5 g per 100 g) and lemon juice. This was prepared several times prior to the final combination. Then, a sensory test was performed with 50 voluntary participants (84% females and 16% males) with a mean age of 21.84 (± 4.55) years, to evaluate the acceptability of both samples. A Food Neophobia Questionnaire (FNQ) and a Samples Assessment Questionnaire (SAQ) were also applied. TAC was quantified by a Folin–Ciocalteu spectrophotometric method [1].

**Results:**

Sample A contains only pepper and salt (14.8 g per 100 g), while sample B presented additional compounds with antioxidant activity. Concerning FNQ and SAQ, the vast majority of participants were willing to try the samples and had considered sample B has a more pleasant smell, taste and texture. Also, sample B presented a higher TAC than sample A (1.5088mg/mL *versus* 0.6182mg/mL).

**Conclusion:**

The healthier version of MM presented lower salt (12.5% less) and had higher TAC than the traditional recipe. Also, this version was organoleptically acceptable. The higher TAC may be explained by the ingredients used. This study reveals that modifications of traditional recipes may increase nutritional value without compromising acceptance.
